# An automated COVID-19 triage pipeline using artificial intelligence based on chest radiographs and clinical data

**DOI:** 10.1038/s41746-021-00546-w

**Published:** 2022-01-14

**Authors:** Chris K. Kim, Ji Whae Choi, Zhicheng Jiao, Dongcui Wang, Jing Wu, Thomas Y. Yi, Kasey C. Halsey, Feyisope Eweje, Thi My Linh Tran, Chang Liu, Robin Wang, John Sollee, Celina Hsieh, Ken Chang, Fang-Xue Yang, Ritambhara Singh, Jie-Lin Ou, Raymond Y. Huang, Cai Feng, Michael D. Feldman, Tao Liu, Ji Sheng Gong, Shaolei Lu, Carsten Eickhoff, Xue Feng, Ihab Kamel, Ronnie Sebro, Michael K. Atalay, Terrance Healey, Yong Fan, Wei-Hua Liao, Jianxin Wang, Harrison X. Bai

**Affiliations:** 1 Department of Diagnostic Imaging, Rhode Island Hospital, Providence, RI 02903 USA; 2 Department of Computer Science, Brown University, Providence, RI 02912 USA; 3 Warren Alpert Medical School of Brown University, Providence, RI 02912 USA; 4 Department of Radiology, Xiangya Hospital, Central South University, Changsha, Hunan 410011 China; 5 Perelman School of Medicine at University of Pennsylvania, Philadelphia, PA 19104 USA; 6 Athinoula A. Martinos Center for Biomedical Imaging, Department of Radiology, Massachusetts General Hospital, Boston, MA 02129 USA; 7 Center for Computational Molecular Biology, Brown University, Providence, RI 02912 USA; 8 Department of Radiology, Brigham and Women’s Hospital, Boston, MA 02115 USA; 9 Department of Biostatistics, Brown University, Providence, RI 02912 USA; 10 Center for Biomedical Informatics, Brown University, Providence, RI 02912 USA; 11 Carina Medical, Lexington, KY 40513 USA; 12 Department of Radiology and Radiological Sciences, Johns Hopkins University, Baltimore, MD 21205 USA; 13 School of Computer Science and Engineering, Central South University, Changsha, China

**Keywords:** Computer science, Radiography

## Abstract

While COVID-19 diagnosis and prognosis artificial intelligence models exist, very few can be implemented for practical use given their high risk of bias. We aimed to develop a diagnosis model that addresses notable shortcomings of prior studies, integrating it into a fully automated triage pipeline that examines chest radiographs for the presence, severity, and progression of COVID-19 pneumonia. Scans were collected using the DICOM Image Analysis and Archive, a system that communicates with a hospital’s image repository. The authors collected over 6,500 non-public chest X-rays comprising diverse COVID-19 severities, along with radiology reports and RT-PCR data. The authors provisioned one internally held-out and two external test sets to assess model generalizability and compare performance to traditional radiologist interpretation. The pipeline was evaluated on a prospective cohort of 80 radiographs, reporting a 95% diagnostic accuracy. The study mitigates bias in AI model development and demonstrates the value of an end-to-end COVID-19 triage platform.

## Introduction

Coronavirus disease 2019 (COVID-19) caused by severe acute respiratory syndrome coronavirus 2 can result in diverse respiratory symptoms ranging from rhinorrhea to severe acute respiratory distress syndrome^1,2^. As of August 3, 2021, the total number of confirmed cases has reached over 197 million and continues to increase globally^3^. The most effective way to contain the pandemic has been the isolation of symptomatic cases with contact tracing^4^, which ultimately depends on the early detection of COVID-19 in individuals. Efficient triage and determination of disease severity for those who are already infected have also been essential to allocate resources and coordinate appropriate treatment plans.

The standard diagnostic test for COVID-19 currently is the reverse transcriptase-polymerase chain reaction (RT-PCR)^5^. However, its shortfalls include potential false-negative results^6,7^, inconsistent diagnostic accuracy over the disease course^8^, and test kit shortages^9^. Supplementing RT-PCR with medical imaging can help mitigate these limitations. For example, chest radiographs (CXR) can be helpful given their low-dose radiation, relative speed, cost efficiency, portability, and accessibility especially in places with limited resources and staff to manage high patient volumes.

Chest radiographs have shown their efficacy in screening COVID-19 and even in predicting the clinical outcomes of COVID-19 patients, including the deterioration of some to critical status^10–12^. While the American College of Radiology does not recommend using CXR interpretations alone to diagnose COVID-19 or assess disease severity^13^, medical imaging can supplement laboratory findings to better inform clinical decision-making. On CXRs, COVID-19 has characteristic patterns, such as diffuse reticular-nodular opacities, ground-glass opacities, and consolidation especially in peripheral and lower zone distributions with bilateral involvement^14,15^. These findings can inform clinicians not only whether a patient is COVID-19 positive, but also how likely and approximately when he or she will be admitted, mechanically ventilated, or even expire^11,16^.

Prior studies have even leveraged artificial intelligence (AI) to predict patient outcomes from CXRs^17–19^, acknowledging deep learning’s automatic feature extraction and image recognition capabilities. However, previously published studies (Supplementary Tables 1 and 2) are limited by their primary reliance on small public datasets that expose them to considerable risk of selection bias without any external testing to evaluate their models’ ability to generalize on unseen data^20^. Additionally, previous studies do not evaluate the tangible value of their models, foregoing opportunities to compare their models’ performance to those of radiologists or evaluate the additive value of their models when used in conjunction with traditional clinical methods. Lastly, these studies have not publicly shared the code to train and test their models, nor the model files that ensue from it, limiting opportunities for external collaborators to validate and extend findings.

This study has three major contributions: (1) the design and evaluation of a diagnosis AI model that addresses notable shortcomings of prior publications, (2) integration with automated image retrieval tools and prognosis AI models to develop a streamlined triage pipeline that delivers accuracy and timeliness of results, and (3) a comparative assessment of its performance against radiologists, especially to discern early disease findings. Together, the study completes a fully automated pipeline that integrates with the hospital’s existing imaging repository to automatically retrieve chest radiographs and examine them for the presence and severity of COVID-19. Given the lack of COVID-19 studies that transform dynamic data feeds into actionable insights for clinical use, a fully automated AI triage pipeline herein can help expedite, standardize, and directly improve COVID-19 patient care.

## Results

### Patient characteristics

A total of 12,776 CXRs acquired from 10,628 patients were used to train and evaluate the diagnosis prediction model, including 2785 CXRs with COVID-19 pneumonia-related findings from patients with confirmed COVID-19 by RT-PCR. COVID-19 prevalence in the Brown-April, External, and Xiangya-February test sets, respectively, were 70.3%, 24.4%, and 32.9%. The mean age in the training, Brown-April, External, and Xiangya-February datasets for the diagnosis model, respectively, was 56.0 ± 21.0, 62.7 ± 17.6, 46.9 ± 23.3, and 65.1 ± 13.9. The mean age in the training, internal testing, and external testing datasets for the prognosis models, respectively, was 54.8 ± 19.5, 54.2 ± 19.1, and 59.2 ± 19.0. Five hundred and fifty out of 2309 patients among the patient cohort used for the severity and progression models had a critical outcome. The median age of critical patients was higher than that of non-critical patients (67 vs. 51 years, *P* < 0.001). The median number of days from CXR acquisition to a patient’s first critical event was 0.63 days with an interquartile range of 2.61 days.

Variance across training and testing datasets are reported for the diagnosis and prognosis models, respectively, in Tables 1 and 2. Excluding sex distribution of COVID-19 positive patients within the Xiangya-February test set, the calculated *P*-values for the diagnosis model indicate the statistically significant variance of patient demographics across the training and external testing datasets (Table 1). Likewise, the reported *P*-values for the prognosis models indicate that the demographic variance across their test datasets is statistically significant. Additionally, among the 14 assessed pathological and comorbidity variables for the prognosis models, five demonstrated statistically significant variance between the internal and external test datasets. These clinical variables include oxygen saturation on room air (*P* < 0.001), white blood cell count (*P* = 0.007), lymphocyte count (*P* < 0.001), c-reactive protein (*P* < 0.001), and cardiovascular disease (*P* = 0.020).

**Table 1:** Demographic variance across diagnosis model test datasets.

	Training vs. Brown-April	Training vs. External	Training vs. Xiangya-February
*Positive PCR Only*			
Sex	0.499	<0.001	0.343
Age	0.079	0.010	<0.001
*Negative PCR Only*			
Sex	<0.001	<0.001	<0.001
Age	<0.001	<0.001	<0.001
*All patients*			
Sex	<0.001	<0.001	<0.001
Age	<0.001	<0.001	<0.001

*P*-values were calculated using ANOVA and two-sample *t*-tests between the training dataset and each testing sample set.

**Table 2:** Demographic and clinical variance across prognosis model test datasets.

	Training vs. External test	Internal test vs. External test
*Demographic data*		
Sex	<0.001	<0.001
Age	<0.001	<0.001
*Clinical data*		
Temperature	**0.317**	**0.392**
O_2_ Saturation on room air	<0.001	<0.001
White blood cell count	<0.001	0.007
Lymphocyte count	<0.001	<0.001
Creatinine	**0.095**	**0.511**
C-Reactive protein	<0.001	<0.001
Cardiovascular disease	0.029	0.020
Hypertension	0.043	**0.059**
COPD	**0.330**	**0.732**
Diabetes	**0.233**	**0.676**
Chronic liver disease	**0.524**	**0.770**
Chronic kidney disease	0.014	**0.703**
Cancer	**0.322**	**0.689**
Human Immunodeficiency Virus	**0.946**	**0.850**

*P*-values were calculated using ANOVA and two-sample t-tests between the training dataset and each testing sample set, with values >0.05 marked in bold.

### Model and overall pipeline performance

**Fig. 1:**
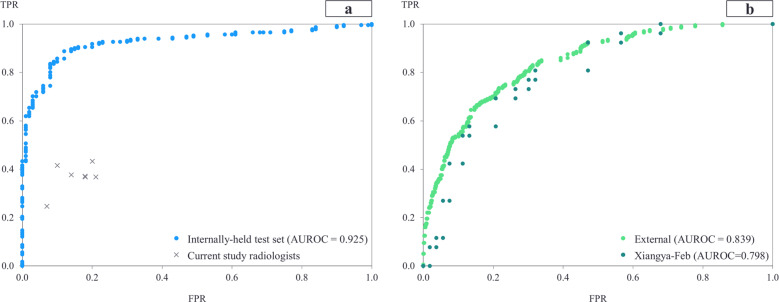
COVID-19 diagnosis AUROC curves for the internally held-out and external test sets. The true and false positive rates for the study’s radiologists are also portrayed to assess model performance relative to traditional clinical methods. **a** Internally held-out test set and **b** external test set. TPR true positive rate, FPR false positive rate.

**Fig. 2:**
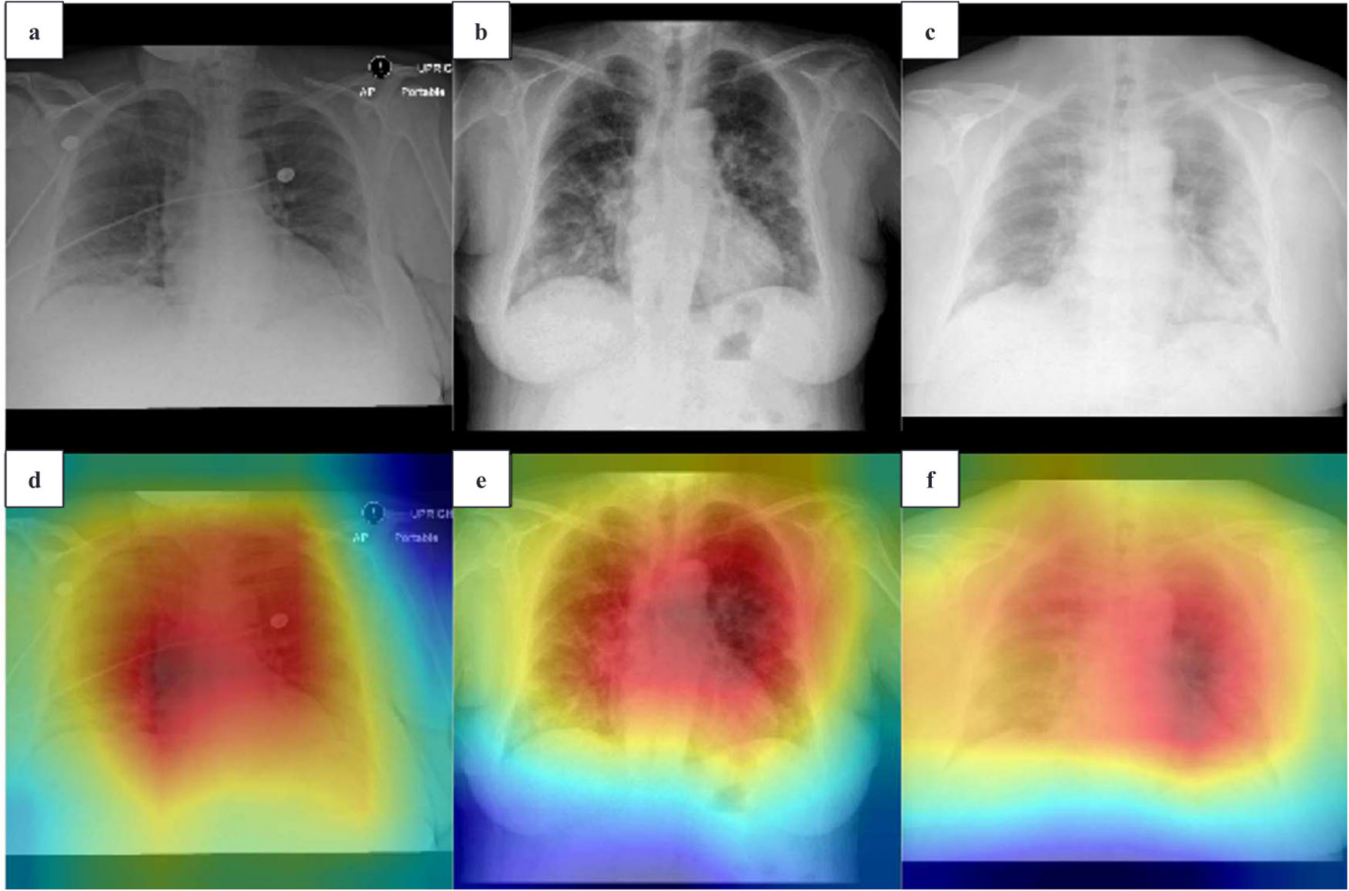
COVID-19 diagnosis model gradient-weighted class activation mapping (Grad-CAM) visualization. All images were predicted correctly as COVID-19 positive. Grad-CAM heatmaps visualize which portions of the input chest radiograph were important for the classification decision. **a** Brown-April, original chest radiographs, **b** External, original chest radiographs, **c** Xiangya-February, original chest radiographs, **d** Brown-April, Grad-CAM overlay, **e** External, Grad-CAM overlay, and **f** Xiangya-February, Grad-CAM overlay.

The diagnosis model achieved an area under the receiver operating characteristic curve (AUROC) of 0.925 internally (Brown-April) and 0.839 and 0.798 externally (External and Xiangya-February) (Fig. 1). On Brown-April, the model was more accurate (accuracy: 77.0% vs. 52.4%; 95% CI: 18.7%, 30.5%; *P* < 0.001), sensitive (sensitivity: 68.3% vs. 38.3%; 95% CI: 22.2%, 37.8%; *P* < 0.001), specific (specificity: 96.6% vs. 84.3%; 95% CI: 5.8%, 19.7%; *P* = 0.020), and balanced (*F*1-score: 80.5% vs. 52.3%, 95% CI: 21.2%, 35.4%; *P* < 0.001) than the average radiologist from the study (Fig. 1). The average radiologist was defined by deriving the mean value for the accuracies, sensitivities, specificities, and *F*1-scores for each of the seven radiologists. Brown-April consisted of 38 CXRs that were marked normal by the original radiology reports despite those patients testing positive via RT-PCR. While this study’s radiologists, respectively, could only label 1 (2.6%), 0, 0, 2 (5.3%), 0, 0, and 1 scans correctly as COVID-19 positive, the model correctly labeled 17 (44.7%) of these scans. Gradient-weighted class activation mapping (Grad-CAM) illustrated that the model recognized lung lesions (Fig. 2), attributing greater input to them when deriving predictions^21^.

The combined severity models reported AUROCs of 0.860 (95% CI: 0.851, 0.866) internally and 0.799 (95% CI: 0.788, 0.810) externally, while the combined progression models reported C-indices of 0.791 (95% CI: 0.786, 0.803) internally and 0.766 (95% CI: 0.753, 0.774) externally. Individually, the image- and clinical-based severity models, respectively, reported AUROCs of 0.814 (95% CI: 0.804, 0.826; *P* < 0.001) and 0.846 (95% CI: 0.837, 0.860; *P* = 0.005) internally and 0.759 (95% CI: 0.746, 0.771; *P* < 0.001) and 0.785 (95% CI: 0.779, 0.799; *P* = 0.005) externally. Meanwhile, the individual image- and clinical-based prognosis models, respectively, reported C-indices of 0.760 (95% CI: 0.746, 0.772; *P* < 0.001) and 0.739 (95% CI: 0.721, 0.748; *P* < 0.001) internally and 0.712 (95% CI: 0.700, 0.719; *P* < 0.001) and 0.718 (95% CI: 0.707, 0.726; *P* < 0.001) externally. As such, leveraging a combination of the image- and clinical-based methods improved model performance.

A total of 820 CXRs collected between October 2020 and November 2020 were processed in real-time using the AI pipeline. The mean latencies for the pipeline and the radiologists, respectively, were 14.3 ± 9.8 and 24.5 ± 28.1 min. Among these studies, 80 CXRs (hereafter referred to as Brown-Autumn) had RT-PCR data acquired within 24 h, 3 (3.8%) of which were COVID-19 positive. The diagnosis model was able to accurately predict 76 (95.0%) of these CXRs for COVID-19 in real-time.

## Discussion

Prompt diagnosis and prognostication of COVID-19 patients can be helpful in containing the pandemic. As hospitals are inundated with COVID-19 patients due to the propagation of new variants, relief of mask and social distancing mandates, and the lack of widespread vaccine adherence, an AI pipeline to effectively triage ED patients can help clinicians better manage limited resources, prepare for adverse events, and maintain a safe environment for staff and other patients. Using AI based on CXRs and clinical data, this study provides an end-to-end solution for COVID-19 diagnosis and prognostication that integrates seamlessly with a hospital’s existing network for immediate clinical use. The pipeline functions as an additional tool that supplements conventional examination methods to triage patients and develop appropriate care plans.

Earlier reviews have published predictive models for COVID-19 diagnosis and prognosis, but many of these studies present numerous sources of potential bias that the proposed study addresses^20^ (Supplementary Discussion). Supplementary Table 1 illustrates a comparative analysis of the current study against previously published COVID-19 diagnostic studies. While the present study seeks to maximize the gains associated with each individual pipeline component, the primary objective was to optimize study design based on notable pitfalls of these previous studies, integrate standalone technologies, and provide a more comprehensive COVID-19 assessment. The goal was not to develop a custom-built convolutional neural network (CNN) architecture for COVID-19 diagnosis and prognosis, hence the models’ usage of EfficientNet, a popular CNN architecture and scaling method well-regarded for its high accuracy and low computational cost^22^. As such, the innovation driven by the present study is not the novelty of algorithms used, but rather by the significant strides taken to enhance the practical utility and clinical adoption of AI-assisted diagnosis and prognosis.

Foremost, most prior studies exclusively utilized public datasets, most notably the COVID-19 Image Data Collection^23^, without validating their models on an external test set. Not only is it often impossible, with public repositories, to confirm that patients are indeed positive for COVID-19 without accompanying RT-PCR results, patient charts, or radiological reports, many of these datasets, including images from the COVID-19 Image Data Collection, have image artifacts that can engender misleading results^20^. In fact, it has been demonstrated that models can learn to “diagnose” COVID-19 with an AUROC of 0.68 from images with lung regions entirely excluded solely from other non-clinical artifacts specific to the institutional source^24^. Many images from public repositories, additionally, are delivered compressed rather than in their original Digital Imaging and Communications in Medicine (DICOM) format. Loss of resolution that is not uniform across classes can lead to model overfitting^20^. While some studies attempt to mitigate bias by segmenting the lung field or visualizing class activation maps, the ability of their models to generalize on unseen data is still conjecture. Prior studies, by neglecting to evaluate their models on an external independent test set, are unable to demonstrate that their models are truly diagnosing for COVID-19, rather than simply identifying the source of the CXR.

Additionally, images that have been extracted from publications and uploaded online are likely to represent more unusual or severe cases of COVID-19. Such overrepresentation can limit a model’s ability to discern preliminary disease findings, reducing the model’s value as a diagnostic tool to detect COVID-19 at an early stage^20^. While the current study leverages public datasets, the authors have also collected a considerably large collection of COVID-19 and non-COVID-19 images of diverse severity and origin, enacting various protocols to ensure that the acquired COVID-19 scans present COVID-19-related pneumonia and are accompanied by timely RT-PCR tests. In fact, the diagnosis model was trained on ~12,000 CXRs, 2360 scans (20.4%) of which manifested COVID-19 pneumonia findings. The remaining scans encompassed diverse thoracic findings, including non-COVID-19 pneumonia, cardiomegaly, lung lesion, lung opacity, edema, consolidation, atelectasis, pneumothorax, and pleural effusion. Without these protocols and by exclusively using public datasets that are limited by their extreme class imbalance, lack of disease severity coverage, and small sample size, prior studies likely have overfitted their models, reporting overly optimistic model performance^20^.

**Fig. 3:**
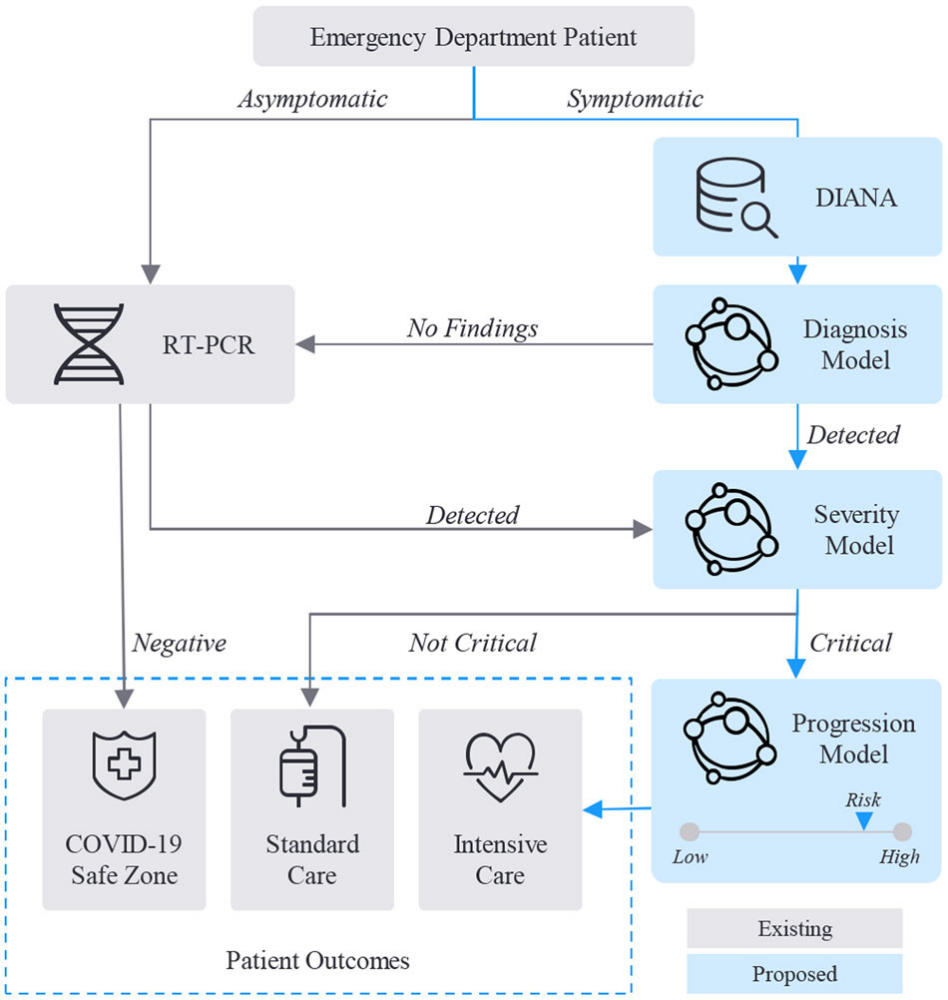
COVID-19 triage pipeline. The blue arrows represent an illustrative example of how a patient presenting with severe COVID-19 and high risk for critical deterioration would be triaged via the automated pipeline. Recommended patient outcomes would require physician approval before execution.

Moreover, previous studies have treated diagnosis and prognosis as isolated problems and have outlined few details on how they can be integrated into an actual clinical workflow. A diagnosis or prognosis model, by itself, lacks the viability to be incorporated for practical use not only because it is only one component of an ecosystem of factors that motivate clinical decision-making, but also because so much human intervention is necessary to utilize them effectively. In fact, traditional methods to access images from the Picturing Archiving and Communication System (PACS) require repetitive, manual querying of the electronic health records, making real-time communication between advanced analytic systems infeasible. To address these gaps, this study utilizes DICOM Image Analysis and Archive (DIANA) to retrieve requested medical images in real-time and pass them as inputs for AI analysis^25^. DIANA uses Docker containerization to easily deploy AI solutions without customized development and acts as a data retrieval engine from the image database. As an end-to-end solution, inputting patient accession numbers would trigger a series of AI models to predict which cases will lead to future COVID-19 complications and hospitalization. Unlike previously published studies (Supplementary Table 2), the prognosis model not only predicts the disease severity of a COVID-19 patient, but also predicts the time until a patient encounters his or her first critical event. By streamlining triage to monitor patient entry into designated COVID-19 safe zones or determine which patients will require standard or intensive care, the study informs hospital personnel with tangible timelines and recommendations to better allocate limited resources and improve patient outcomes. An illustrative workflow is outlined in Fig. 3 to demonstrate how the pipeline can handle different permutations of patient symptoms, CXR presentations, and disease severities.

In addition, the present pipeline can handle a realistic influx of patients into any emergency department (ED). The diagnostic model, for instance, reported a 95% accuracy on Brown-Autumn, a series of CXRs that were collected in real-time from the ED, and was tested on two external test sets to assess its ability to generalize on unseen data. While some performance loss was noted between the internal and external test sets (diagnosis AUROC: 0.925 versus 0.839 and 0.798; severity AUROC: 0.860 versus 0.799; prognosis concordance index [C-index]: 0.791 versus 0.766), imperfect generalization is expected given patient populations that are inevitably unrepresented and the inconsistent image acquisition conditions across institutions, including variability of equipment, techniques, and operators. In fact, analysis of variance (ANOVA) and two-sample *t*-tests attest to statistically significant demographic variance between the training and external tests for both the diagnosis and prognosis models. Despite these differences, the system continues to accurately predict COVID-19 diagnosis, severity, and time-to-event progression, supporting the model’s ability to generalize on unseen populations and new institutions. This represents a stark contrast to previous studies that evaluate their models solely on internal test sets, whose patient demographic distribution likely resembles that of their training dataset as seen with Brown-April in Table 1. This evaluation encourages model overfitting, likely contributing to overly optimistic model performance and the lack of practical utility as the model cannot be adopted for widespread clinical use.

As such, the exposure to a wide gamut of CXR presentations likely enhanced the robustness of this study’s prediction models to continue operating effectively on different hospital networks without customizing the model design or significantly re-tuning model parameters for each institution. Image preprocessing techniques to standardize CXRs and mitigate data harmonization concerns enable the platform to be a fully scalable solution that can be deployed within a reasonable timeframe for most hospital institutions. Foregoing this process not only lengthens implementation timelines but also limits model accessibility to large multi-institution networks as smaller hospitals may not have the technical infrastructure or sufficient influx of patients to provide a meaningful sample of radiographs for hypothetical model re-tuning.

**Fig. 4:**
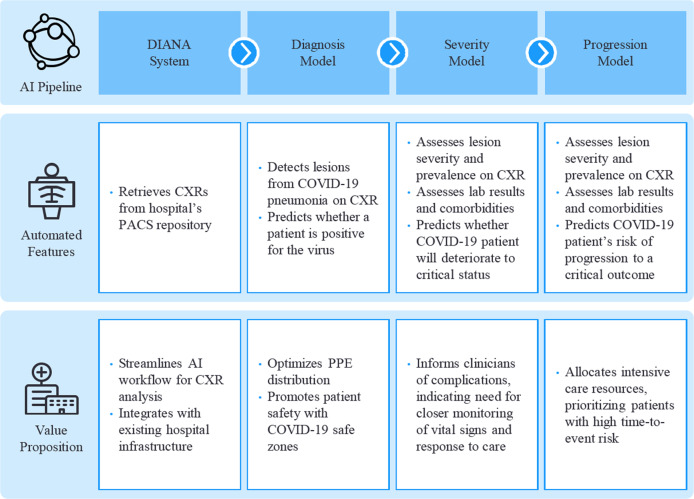
Features and value propositions of individual pipeline components. The outlined components work together to deliver an end-to-end pipeline to rapidly identify and triage COVID-19 patients within an emergency department. Tangible value propositions are outlined for each component.

Finally, the study illustrates the impressive pattern recognition ability of deep learning methods. The diagnosis model outperformed human evaluators in its ability to detect COVID-19 from CXRs with statistical significance (*P* < 0.05). Furthermore, the model correctly flagged 17 of 38 (44.7%) CXRs that were originally marked as normal by the original radiologist, despite having been acquired from patients with confirmed COVID-19 via RT-PCR. In contrast, most radiologists from this study were likewise unable to detect indications of COVID-19 from these scans. This outcome adds to the increasing evidence that nascent COVID-19 findings can be difficult to discern. Especially as misdiagnosis can stem a series of misinformed decisions as care plans often commence with a diagnosis, the proposed pipeline can contribute immense value as an auxiliary tool to supplement conventional examination. Figure 4 summarizes the key features and value proposition of each component.

This study has several limitations that warrant further investigation. First, the present study utilized a training set of chest scans acquired only from the ED. The current inclusion and exclusion criteria thus introduce some selection bias, as they do not consider patients from outpatient services or those who encountered a critical event from a subsequent admission after being discharged. Among these scans, the CXRs from patients with confirmed COVID-19 used to train the diagnosis model regarding the positive class were largely from one institution. While class activation mapping was employed to verify that the model was not learning medically irrelevant differences between data subsets, the training dataset can be further improved by including more COVID-19 negative CXRs from this hospital network and by expanding the diversity of CXR sources for the positive class. Additionally, RT-PCR results were used as ground-truth labels for the diagnosis model, despite their limited sensitivity, given the expansive size of the training set. A future study that monitors patients presenting to the ED with respiratory or flu-like symptoms and evaluates the pipeline against several RT-PCR results throughout the patient’s participation could mitigate this limitation and assess the model’s true accuracy. Last, further investigation is necessary to confirm the interpretability of the pipeline’s outputs in an actual clinical setting and quantitatively measure its incremental value in improving patient outcomes. The study at hand is primarily a technological proof of concept that optimizes and integrates standalone technologies to lay the foundation for future studies, including those that enhance the model’s interpretability for non-technical users within the healthcare community. While AI-assisted diagnostics and prognosis may enhance efficiency and accuracy, further development to increase its ease-of-use, such as an intuitive low-code/no-code front-end and auto-generated descriptions to explain AI output, is necessary to augment the model’s acceptance and adoption across a wide assortment of clinical staff.

The study addresses one of the key issues in AI research—its practical implementation for clinical use. By addressing major shortcomings of prior publications and developing a fully automated pipeline to retrieve chest radiographs and examine them for the presence and severity of COVID-19 pneumonia, the authors provide a framework that leverages deep learning solutions to expedite triage and inform clinical decision-making with data-driven insights. This AI pipeline is designed to be utilized as an additional tool to supplement and enhance conventional examination for COVID-19 triage, rather than replacing it altogether. Upon any CXR acquisition, the system would retrieve the relevant image, patient, and clinical data via DIANA and feed the appropriate inputs into the respective AI models for diagnosis and prognosis. As illustrated in Fig. 3, each component helps the integrated pipeline triage a patient into three likely outcomes, depending on the presence and severity of COVID-19 pneumonia. The results can be employed as an initial screening tool within the emergency department to flag patients requiring immediate attention or imminent life-supporting resources, such as respiratory ventilators. A system that can quickly deliver preliminary findings of the status and anticipated care a patient will require can help clinicians prepare for and address complications earlier. The results can also be used as a confirmatory second opinion to validate initial radiological findings via traditional examination or to identify abnormal lung regions that may have been difficult to discern without AI assistance. As such, the tool is designed to be used alongside traditional methods for patients presenting to the emergency room with respiratory symptoms and requiring a CXR. Specific timelines will likely vary, but the inherent nature of an integrated pipeline to be fully automated allows hospitals to determine when and how the tool will be used, whether that be for emergency screening, confirmatory assessments, or fail-safe checks. The present study, therefore, validates the feasibility and value of having an end-to-end AI platform that expedites and enhances traditional examination methods.

## Methods

### Data collection and cleaning

**Fig. 5:**
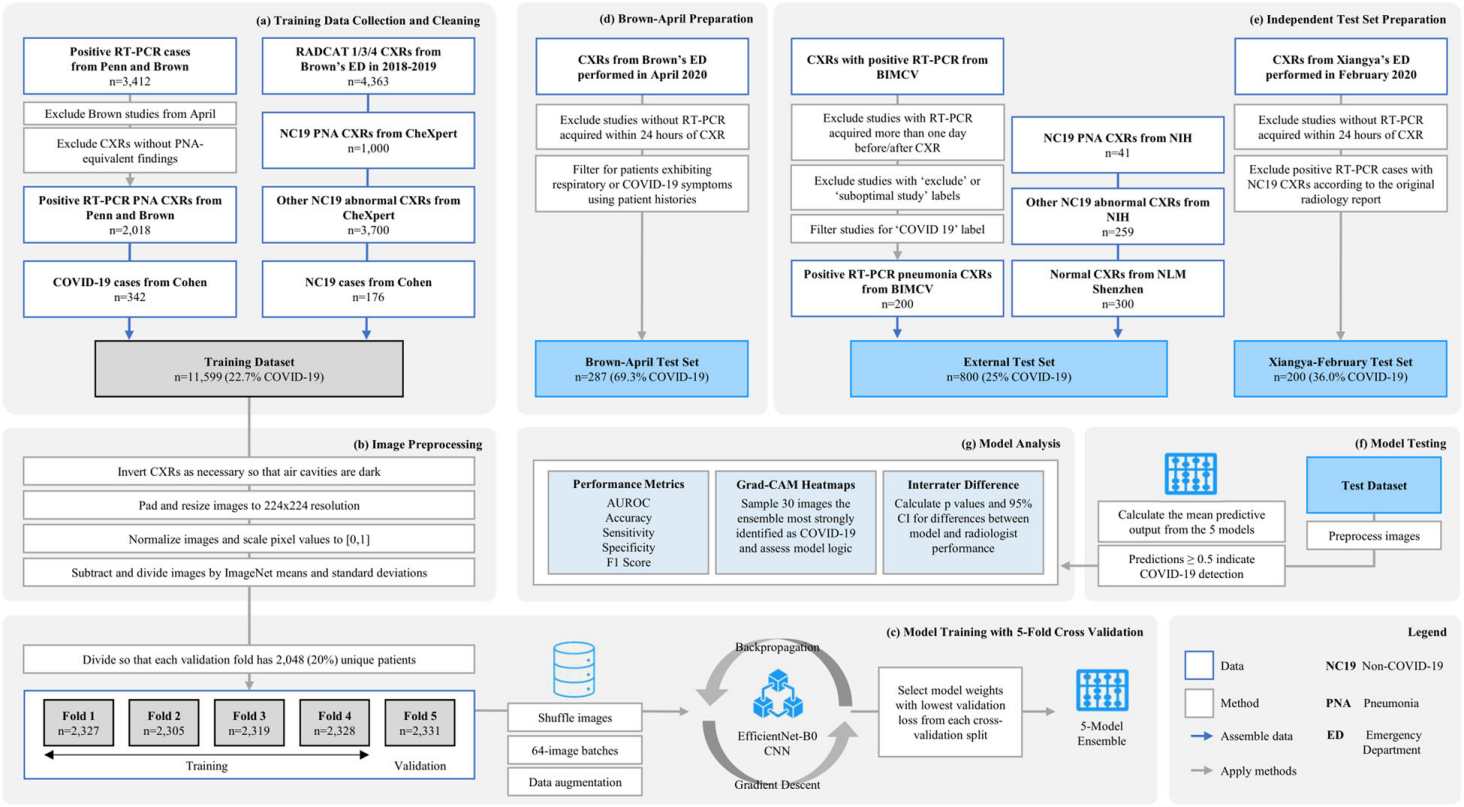
COVID-19 diagnosis prediction model development. The flowchart delineates the inclusion and exclusion criteria for the training and testing cohorts, as well as the methods to train, test, and evaluate the model.

A collection of 7775 CXRs were retrieved from the ED of four hospitals affiliated with the University of Pennsylvania Health System (Penn) in Philadelphia, Pennsylvania, and four hospitals affiliated with Brown University (Brown) in Providence, Rhode Island. Among this cohort, 3412 CXRs were acquired between February 2020 and July 2020 from patients with confirmed COVID-19 via RT-PCR (COVID-19 RT-PCR test from Laboratory Corporation of America). As some RT-PCR results were dated more than 24 h apart from the CXR acquisition, the radiology report was used to determine if each CXR from patients with confirmed COVID-19 via RT-PCR manifested pneumonia. Only the 2018 pneumonia-presenting CXRs, 1774 of which were acquired within a day of RT-PCR administration, were utilized for the positive class to train the diagnosis model. Asymptomatic cases were excluded to permit model convergence for COVID-19 positive detection from CXRs. Pneumonia CXRs dating before December 2019 were used to train for the negative class so that the model could discern between pneumonia of COVID-19 and other viral/bacterial etiologies. The distribution of COVID-19 and non-COVID-19 cases, as well as certain exclusion criteria, of the internally held-out and two independent external test sets for the diagnosis model, are illustrated in the fourth step, “Brown-April preparation” and “Independent test set preparation,” of Fig. 5. The prognosis models for severity classification and time-to-event prediction were trained using a 7:1:2 train-validation-split on the CXRs from the 2011 Penn patients. Chest radiograph images from Brown patients were not utilized to train the prognosis models.

The remaining 4363 CXRs the authors collected were acquired between January 2018 and December 2019 and were from uninfected patients. This COVID-19 negative subset consisted of 3301 RADCAT 1, or normal, and 1062 RADCAT 3 and 4, or urgent and priority, scans^26^. RADCAT is a structured reporting system, through which radiologists can assign medical images a score ranging from 1 (normal) to 5 (critical) to categorize and communicate findings more easily. The entire dataset was supplemented with 518 CXRs, of which 342 scans presented COVID-19 findings, from the COVID-19 Image Data Collection^23^ and 4700 non-COVID-19 CXRs from CheXpert^27^, a library of CXRs acquired before July 2017 from Stanford Hospital. All scans were either in the posterior-anterior or anterior-posterior view. The methodology behind training dataset construction for the diagnosis model is illustrated in the first step, “Training data collection and cleaning,” of Fig. 5.

The Brown cohort of ~5000 scans was automatically retrieved from the hospitals’ PACS using DIANA. This system uses open-source software, such as the Docker container system and the Orthanc lightweight DICOM server, to deploy replicable image retrieval scripts on an institutional machine. At a high level, DIANA uses containerized Orthanc instances to communicate with PACS and programmatically retrieve anonymized images. Images are processed on an AI container and presented to end-users on a communications platform of choice^25^.

Brown CXRs from April 2020 was held out as an internal test set (Brown-April) for the diagnosis model. Brown-April consisted of 287 scans, 199 of which were from patients with confirmed COVID-19 via RT-PCR and exhibiting respiratory symptoms. Brown-April CXRs were independently evaluated for the presentation of COVID-19 pneumonia by seven radiologists, respectively, with 15, 3, 1, 5, 3, 6, and 2 years of experience examining CXRs. While the patient age and sex were provided, image properties were removed by preprocessing, shuffling, renaming, and resizing scans.

Two datasets (Xiangya-February and External) were used for external testing of the diagnosis model. Xiangya-February consisted of 200 scans from the Xiangya Hospital of Central South University in Changsha, China, 72 scans of which were collected from 79 patients with confirmed COVID-19 via RT-PCR and exhibiting respiratory symptoms. External was compiled from public repositories, including the Valencian Region Medical ImageBank COVID-19+ dataset^28^, the National Institute of Health^29^, and the Shenzhen Hospital CXR dataset^30^. This compilation consisted of 200 scans with COVID-19 pneumonia-related lesions, 300 scans with non-COVID-19 findings, and 300 scans without findings. All scans were collected within 24 h of RT-PCR acquisition. The methodologies to construct the external test sets for the diagnosis model, as well as their proportions of COVID-19 images, are outlined in the fourth step, “Brown-April preparation” and “Independent test set preparation,” of Fig. 5. CXR scans from 546 Brown patients were compiled to assemble an external test set for the prognosis models.

The retrospective study was conducted in accordance with the Declaration of Helsinki and was approved by the Institutional Review Boards of all participating hospital institutions. Image data were deidentified, and personal health information was anonymized. External study sponsors were not involved in the study design; collection, analysis, and interpretation of data; writing of the report; nor the decision to submit the paper for publication. All authors had full access to the data in the study and accepted responsibility for the content herein to submit for publication.

### Predictive AI model development

All images were downloaded at their original dimensions and resolution. Images downloaded in DICOM format were inverted, if necessary, and saved as PNG files. All images were padded, uniformly resized, and converted into 3-channel data. The images were rescaled and normalized using the channel-wise ImageNet means and standard deviations^31^. Preprocessing the images helped address data harmonization concerns across multiple datasets, standardizing the images to provide a comparable view of data across sources.

Diagnosis models were developed using EfficientNet-B0 models initialized on ImageNet pretrained weights^31^ and trained using 5-fold cross-validation without patient overlap between folds (Fig. 5). Models were trained using the Adam optimizer^32^, sigmoid activation, and weighted binary cross-entropy loss to update their weights. Models with the lowest validation losses were selected to minimize overfitting. Figure 5 delineates the methodology to train, validate, and test the diagnosis model. The subsequent severity and progression prediction models employed a likewise workflow, navigating training data collection and cleaning, image processing, model training with cross-validation, external test set preparation, model testing, and model analysis^19^.

Severity models were developed to predict from the segmented CXR images and clinical data whether a patient would encounter a critical event^19^. Lung regions were automatically segmented using a U-Net model^33^ that employed a pretrained VGG-11 feature extractor. An EfficientNet-B0 model initialized on ImageNet pretrained weights^31^ was used to extract features from the masked scan. The output was passed to four prediction layers—one convolutional layer (256) with global average pooling followed by three dense layers (256, 32, 2). An adjunct model comprising three dense layers (16, 32, 2) used 16 demographic, pathology, and comorbidity variables to also predict disease severity. The weighted sum between the image-based and clinical-based predictions was used to inform whether the disease severity was critical.

Time-to-event progression models were developed to predict a COVID-19 patient’s risk of deterioration to their first critical outcome^19^. The CXR features from one of the severity model’s dense layers (256) and the 16 clinical variables were passed as respective inputs to the image-based and clinical-based survival forest models. The weighted sum of the image-based and clinical-based predictions assessed how likely, and approximately when, a patient would deteriorate to his or her first critical outcome.

### Statistical analysis and pipeline evaluation

Variance across training and testing datasets was measured using ANOVA for binary variables and two-sample *t* tests for continuous variables. A *P*-value smaller than 0.05 was interpreted as the means across samples being significantly different. The diagnosis prediction model was evaluated (Fig. 5) by its AUROC against the RT-PCR results on the internal and external test sets. The algorithm’s performance was compared to those of seven board-certified radiologists. *P*-values and 95% confidence intervals were obtained for the interrater differences using the bootstrap method^34^. The 95% confidence intervals of AUROC were determined for the severity model using the adjusted Wald method^35^. The C-index for right-censored data was calculated to evaluate the performance of the progression prediction models^36^.

The pipeline was integrated within Rhode Island Hospital’s network and electronic health record system to evaluate queued CXRs from the ED in real-time. The accuracy of the system was noted, and the latency of the platform was compared to that of the radiologists. The latency of the latter group was defined as the time between CXR acquisition and report creation.

### Ethics disclaimers

The retrospective study was conducted in accordance with the Declaration of Helsinki and was approved by the Institutional Review Boards of all participating hospital institutions. Image data were deidentified, and personal health information was anonymized. External study sponsors were not involved in the study design; collection, analysis, and interpretation of data; writing of the report; nor the decision to submit the paper for publication. All authors had full access to the data in the study and accepted responsibility for the content herein to submit for publication.

### Reporting summary

Further information on research design is available in the Nature Research Reporting Summary linked to this article.

## Supplementary information

Supplemental Information

Reporting Summary

## Data Availability

The chest radiographs the authors collected and used for this study are not available for public access due to data privacy and patient confidentiality clauses governed by HIPPA regulations. Limited data access is obtainable upon reasonable request by contacting the corresponding author.

## Code availability

Model files and code for the AI prediction models within the triage pipeline are available in the following repositories: https://github.com/chrishki/COVID19CXR and https://github.com/ZhichengJiao/COVID-19-prognostic-model.
