# Supplementary material for: An automated COVID-19 triage pipeline using artificial intelligence based on chest radiographs and clinical data
Source: NPJ Digit Med. 2022 Jan 14;5:5. doi: 10.1038/s41746-021-00546-w (PMC8760275; doi:10.1038/s41746-021-00546-w)
Supplement: Supplementary file 1 — Supplemental Information [file 41746_2021_546_MOESM1_ESM.pdf]

Supplementary Table 1

Reference publication	Training and validation				Evaluation				
	Training and valid-ation scans (COVID scans)[1]	Authors collect original COVID scans for training	Authors report timing between CXR and RT-PCR	Internal 5-fold CV[1]	Internal holdout test set[1]	Internal testing scans (COVID scans)[1]	Authors assess generaliz-ability with external testing	Authors compare model perform-ance to that of a radiol-ogist	Authors publicly share code with trained model[1]
Current study	11,599 (2,360)	✓	✓	✓	✓	287 (199)	✓ 200 (72 COVID-19) scans; 800 (200 COVID-19) scans	✓	✓
Li, X. et al.[1]	429 (143)	✗	✗	✗	✓	108 (36)	✗	✗	✓
Luz, E. et al.[2]	13,569 (152)	✗	✗	✗	✓	231 (31)	✗	✗	✓
Bassi, P. and Attuz, R.[3]	2,724 (159)	✗	✗	✗	✓	180 (60)	✗	✗	✗
Heidari, M. et al.[4]	8,474 (415)	✗	✗	✗	✓	848 (42)	✗	✗	✗
Zhang, R. et al.[5]	5,236 (2,582)	✓	✓	✗	✓	5,869 (3,223)	✗	✓	✗
Zhang, R. et al.[6]	386 (150)	✗	✗	✗	✓	101 (39)	✗	✗	✗
Wang, Z. et al.[7]	3,522 (204)	✗	✗	✗	✓	61 (20)	✗	✓	✗
Tsiknakis, N. et al.[8]	458 (98)	✗	✗	✓	✗	114 (24)	✗	✗	✗
Malhotra, A. et al.[9]	24,724 (348)[1]	✗	✗	✗	✓	6,174 (125)[1]	✗	✗	✗
Rahaman, M. et al.[10]	720 (220)	✗	✗	✗	✓	140 (40)	✗	✗	✗
Tamal, M. et al.[11]	378 (226)	✗	✗	✗	✓	165 (115)	✗	✗	✗

[1] These columns are from a published comparative study[15] except for Malhotra et al.[9], where figures have been updated to reflect the actual number of COVID-19 training images before augmentation. Studies that have been withdrawn, that did not indicate sample sizes for development and testing, or that did not report model performance have been excluded.

Differentiation of proposed study: Diagnosis. The table above compares the current study to other published papers that have developed and evaluated machine learning models for COVID-19 diagnosis from CXRs. *CV*, cross-validation; *CXR*, chest x-ray; *RT-PCR*, reverse transcription polymerase chain reaction; *COVID*, coronavirus disease 2019

Supplementary Table 2

Reference publication	Prognosis type[1]	Training and validation COVID sample size[1]	Authors collect original COVID scans for training	Internal holdout test set (COVID scans)[1]	Authors assess generalizability with external testing? (COVID scans)[1]	Authors publicly share code with trained model[1]
The current study	Severity and time to first critical event	1,468 patients	✓	✓ (366 patients)	✓ (475 patients)	✓
Li, M. et al.[12]	Severity	354 scans	✓	✓ (108 scans)	✓ (111 scans)	✗
Li, M. et al.[13]	Severity	314 scans	✓	✓ (154 scans)	✓ (113 scans)	✗
Cohen, J. P. et al.[14]	Lung opacity and extent of lung involvement with grand glass opacities	47 patients	✗	✓ (47 patients)	✗	✓

[1] These columns are from a published comparative study[15]. Studies that have been withdrawn, that did not indicate sample sizes for development and testing, or that did not report model performance have been excluded.

Differentiation of proposed study: Prognosis. The table above compares the current study to published papers that have developed and evaluated machine learning models for COVID-19 prognosis from CXRs. *CV*, cross-validation; *CXR*, chest x-ray; *RT-PCR*, reverse transcription polymerase chain reaction; *COVID*, coronavirus disease 2019

Supplemental Discussion

Several notable differences between the proposed and prior studies are the sheer size of the study's training and validation sample (Supplementary Tables 1 and 2), including the COVID-19 images, relative to prior studies, as well as the procedures to mitigate sources of bias, including:

Diversity of disease origins, severities, and complexities

Original images were collected from an actual influx of ED patients, contributing to a diverse dataset of clinical findings that represent a real-life distribution of disease origins, severities, and complexities. Most prior studies (Supplementary Tables 1 and 2), however, relied on public repositories, which often are pieced together to represent an unrealistic sample and often exhibit an overrepresentation of severe disease cases as unusual or severe presentations are more likely to be uploaded online.

RT-PCR timing with CXR

Radiology reports were leveraged in conjunction with RT-PCR results to detect COVID-19 pneumonia, increasing the confidence that images within the positive class indeed demonstrate COVID-19 pneumonia-related findings. Additionally, the authors considered the timing between CXR acquisition and RT-PCR administration to confirm the validity of ground truth labels. Almost 90% of original CXR scans within the training set were collected within one day of RT-PCR administration, while all CXRs within the test sets were collected within 24 hours of RT-PCR administration. Most prior studies relied on public repositories, which often assign binary values with no supporting documentation, such as RT-PCR data, radiology reports, or patient charts, to validate these findings. As there are often no restrictions for contributors to share COVID-19 CXRs to public datasets, there is no guarantee that positive cases indeed represent COVID-19 disease findings[15].

Selection bias mitigation via cross-validation

The study employed 5-fold cross-validation to minimize selection bias and increase its sample size for training and validation. As such, the model comprises an ensemble of five models tested on a unique outside fold, mitigating the likelihood of selecting a fortuitously favorable internal validation set. This technique, as demonstrated in Supplementary Table 1, was not observed in prior studies, exposing them to additional sources of potential bias.

Multiple external test sets

The ability for the model to generalize was evaluated on various external test sets for the diagnosis and prognosis components of the triage pipeline. The study demonstrates that the pipeline can accurately output predictions on unseen data and is not overfitted to the training data. As prior studies often do not employ external testing to assess model generalizability, they are likely subject to overfitting and overly optimistic results for two notable reasons. First, most prior studies were trained on public repositories that are likely to have more severe cases of COVID-19, impairing their models' ability to detect early stage disease findings. Second, prior studies mostly relied on the COVID-19 Image Data Collection, which has been demonstrated to exhibit distinct image artifacts[16]. Even with preprocessing techniques, such as lung segmentation, and visualization techniques, such as Grad-CAM, it is impossible to fully discern whether a

given model is basing predictions from actual COVID-19 findings or inherent image artifacts without external validation.

Performance comparison to radiologists

The authors compared the performance of the models to those of radiologists. The authors demonstrate that the diagnosis model was able to outperform the average radiologist by a statistically significant margin and correctly detect COVID-19 from 17 of 38 CXRs that were originally marked as normal by the original radiologist, as well as most radiologists from the study. The study, thus, addresses the increasing evidence that COVID-19 at an early stage can be difficult to discern, exemplifying the value of an AI solution in actual clinical workflows.

Public code and model sharing

The authors have published their code (Supplementary Tables 1 and 2), as well as the trained models they have developed. By publicly sharing the code and model files, as well as deploying the prediction models as web applications, the authors invite other researchers to replicate the study's findings and share their advancements in medical image analysis.

While the study leverages several well-known deep learning methodologies to develop an automated pipeline for rapid triage of COVID-19 patients, the authors have designed a study that has addressed notable risks of biases from prior studies that comprise data integrity and clinical viability of their proposed models. Combined with the value of its associated dataset, the proposed AI and informatics pipeline has immense value as a clinical tool that can streamline COVID-19 triage and improve patient outcomes.

Supplementary References

1. Li, X. et al. COVID-MobileXpert: on-device COVID-19 screening using snapshots on chest X-rays. Preprint at http://arxiv.org/ abs/2004.05717 (2020).
2. Luz, E. et al. Towards an effective and efficient deep learning model for COVID-19 patterns detection in X-ray images. Preprint at http://arxiv.org/ abs/2004.05717 (2020).
3. Bassi, P. and Attuz, R. A deep convolutional neural network for COVID-19 detection using chest X-rays. Preprint at http://arxiv.org/ abs/2005.01578 (2020).
4. Heidari, M. et al. Improving the performance of CNN to predict the likelihood of COVID-19 using chest X-ray images with preprocessing algorithms. *International Journal of Medical Informatics* **144,** 104284. https://dx.doi.org/10.1016%2Fj.ijmedinf.2020.104284 (2020).
5. Zhang, R. et al. Diagnosis of COVID-19 pneumonia using chest radiography: value of artificial intelligence. *Radiology* **298,** E88-E97 (2020).
6. Zhang, R. et al. COVID19XrayNet: a two-step transfer learning model for the COVID-19 detecting problem based on a limited number of chest X-ray images. *Interdisciplinary Sciences: Computational Life Science* **12,** 555-565 (2020).
7. Wang, Z. et al. Automatically discriminating and localizing COVID-19 from community-acquired pneumonia on chest X-rays. *Pattern Recognition* **110,** 107613 (2021).
8. Tsiknakis, N. et al. Interpretable artificial intelligence framework for COVID-19 screening on chest X-rays. *Experimental and Therapeutic Medicine*. **20,** 727-735 (2020).
9. Malhotra, A. et al. Multi-task driven explainable diagnosis of COVID-19 using chest X-ray images. Preprint at https://arxiv.org/abs/2008.03205 (2020).
10. Rahaman, M. et al. Identification of COVID-19 samples from chest X-ray images using deep learning: a comparison of transfer learning approaches. *Journal of X-Ray Science and Technology* **28,** 821-839 (2020).
11. Tamal, M. et al. An integrated framework with machine learning and radiomics for accurate and rapid early diagnosis of COVID-19 from chest X-ray. Preprint at medRxiv https://doi.org/10.1101/2020.10.01.20205146 (2020).
12. Li, M. et al. Improvement and multi-population generalizability of a deep learning-based chest radiograph severity score for COVID-19. Preprint at medRxiv https://doi.org/10.1101/2020.09.15.20195453 (2020).
13. Li, M. et al. Automated assessment and tracking of COVID-19 pulmonary disease severity on chest radiographs using convolutional siamese neural networks. *Radiology Artificial Intelligence* **2,** e200079 (2020).
14. Cohen, J. P. et al. Predicting COVID-19 pneumonia severity on chest X-ray with deep learning. Preprint at https://arxiv.org/abs/2005.11856 (2020).
15. Roberts, M. et al. Common pitfalls and recommendations for using machine learning to detect and prognosticate for COVID-19 using chest radiographs and CT scans. *Nature Machine Intelligence* **3,** 199-217 (2021).
16. Maguolo, G. and Nanni, L. A critic evaluation of methods for COVID-19 automatic detection from X-ray images. Preprint at http://arxiv.org/ abs/2004.12823 (2020).
